# Biased Quantification of Rat Liver Fibrosis—Meta-Analysis with Practical Recommendations and Clinical Implications

**DOI:** 10.3390/jcm12155072

**Published:** 2023-08-01

**Authors:** Patrik Mik, Katsiaryna Barannikava, Polina Surkova

**Affiliations:** 1 Department of Anatomy, Faculty of Medicine in Pilsen, Charles University, alej Svobody 76, 323 00 Pilsen, Czech Republic; 2 Biomedical Center and Department of Histology, Faculty of Medicine in Pilsen, Charles University, alej Svobody 76, 323 00 Pilsen, Czech Republic

**Keywords:** biopsy, collagen proportionate area, connective tissue, fibrosis, liver, quantification, stereology

## Abstract

For liver fibrosis assessment, the liver biopsy is usually stained with Masson’s trichrome (MT) or picrosirius red (PSR) to quantify liver connective tissue (LCT) for fibrosis scoring. However, several concerns of such semiquantitative assessments have been raised, and when searching for data on the amount of LCT in healthy rats, the results vastly differ. Regarding the ongoing reproducibility crisis in science, it is necessary to inspect the results and methods, and to design an unbiased and reproducible method of LCT assessment. We searched the Medline database using search terms related to liver fibrosis, LCT and collagen, rat strains, and staining methods. Our search identified 74 eligible rat groups in 57 studies. We found up to 170-fold differences in the amount of LCT among healthy Wistar and Sprague–Dawley rats, with significant differences even within individual studies. Biased sampling and quantification probably caused the observed differences. In addition, we also found incorrect handling of liver fibrosis scoring. Assessment of LCT using stereological sampling methods (such as systematic uniform sampling) would provide us with unbiased data. Such data could eventually be used not only for the objective assessment of liver fibrosis but also for validation of noninvasive methods of the assessment of early stages of liver fibrosis.

## 1. Introduction

The distribution of fibrous connective tissue in the healthy liver provides an anchoring site for the spatial positioning of liver cells and the liver vascular tree with associated autonomic nerves, and mediates spatially organized signaling to the cells. However, pathological excessive secretion of extracellular matrix proteins—including their major components, collagens—distorts the liver architecture, which precludes hepatocytes from their physiological functioning and limits intrahepatic blood flow conditions. This limit leads to hepatic insufficiency, which can progress through liver fibrosis to liver cirrhosis and liver failure. Although several noninvasive scores for staging and grading liver fibrosis have been developed, histopathological evaluation of the liver biopsy is still necessary in assessing hepatic disease and is the most accurate tool in the assessment of liver fibrosis and hepatic disease prognosis [1].

After histological processing, the liver biopsy is usually stained with hematoxylin and eosin, and for the scoring of liver fibrosis, either with picrosirius red (PSR) or with Masson’s trichrome (MT) or both. To assess liver fibrosis, one of the six scoring systems for the assessment of liver fibrosis is usually used (Table S1), with four to eight stages of liver fibrosis and cirrhosis being the last stages. Generally, the actual stage of liver fibrosis is the result of both the amount and the architecture of liver connective tissue (LCT) that both surrounds the liver lobule and forms within the liver lobule (enlarged portal tracts, forming of septa, bridging, and cirrhotic nodules).

Human liver experiments are, however, restricted due to ethical measures. Therefore, among others, rat liver models were established. For any experiment regarding histological quantification of liver fibrosis, reference group data are necessary. Unfortunately, when searching for consensus on the amount of healthy rat LCT, none is to be found. On the other hand, a reliable and valid assessment of LCT provides us with reproducible data for experimental planning, for observational studies, and for the assessment of noninvasive methods of liver fibrosis. Regarding the ongoing crisis in replicating experimental results throughout scientific disciplines [2], it is necessary to scrutinize the results and methods of rat LCT quantification, and to provide guidelines for LCT quantification. Furthermore, such unbiased data could eventually be used not only for the objective assessment of liver fibrosis, but also for the validation of noninvasive methods of the assessment of early stages of liver fibrosis.

## 2. Materials and Methods

This meta-analysis was conducted following the Preferred Reporting Items for Systematic Reviews and Meta-analyses statement [3].

### 2.1. Eligibility Criteria

**Figure 1:**
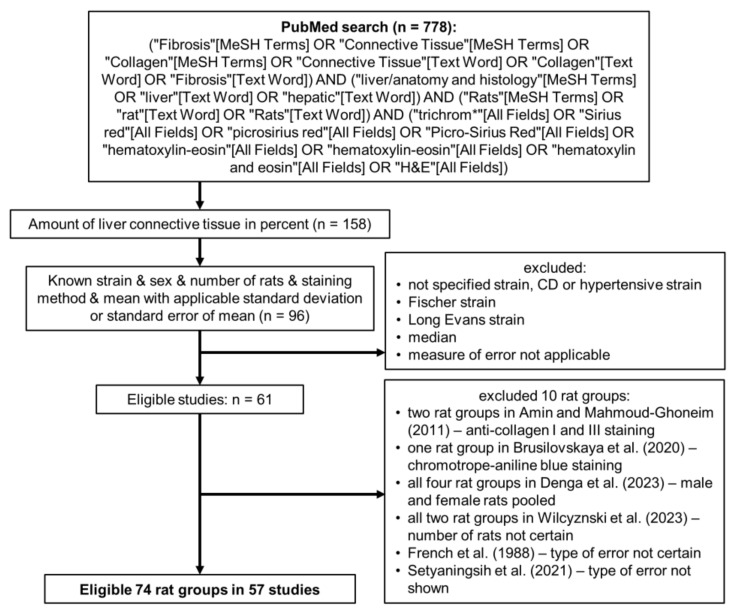
Diagram detailing the literature search. Additionally excluded rat groups were from studies [4,5,6,7,8,9].

We included all studies estimating the mean amount of LCT in healthy Sprague–Dawley and Wistar rats, providing the quantification of the connective tissue on histological specimens stained with MT or PSR. The amount of LCT in the studies was required to be presented as a mean in percent with standard deviation or error, either graphed or displayed as a number. Where the data were graphed, we included only the studies with graphs with clearly visible error bars. In addition, information about sex and the number of individuals had to be provided (see Figure 1).

### 2.2. Search Strategy

We searched the PubMed database using search terms related to liver fibrosis, LCT and collagen, rat strains, and staining methods, without any restrictions on time or study design or types of articles (Figure 1). However, we searched only studies written in English. The search was performed between January and March 2022, and in May 2023. All the studies found through the primary search were reviewed, and only those that matched our criteria were selected for inclusion. Where multiple eligible rat groups were researched in one study, we included all the eligible groups. Retrieved data were collected into a spreadsheet that included bibliographic information, rat strain, rat sex, number of animals, staining method, mean amount of connective tissue with measure of variability (standard deviation of error), and type of presented data (graphed or text). For unbiased assessment of possible differences, we grouped the rats according to their strain, sex, and the method of staining. This resulted in five separate analyses of variances.

### 2.3. Statistical Analysis

**Figure 2:**
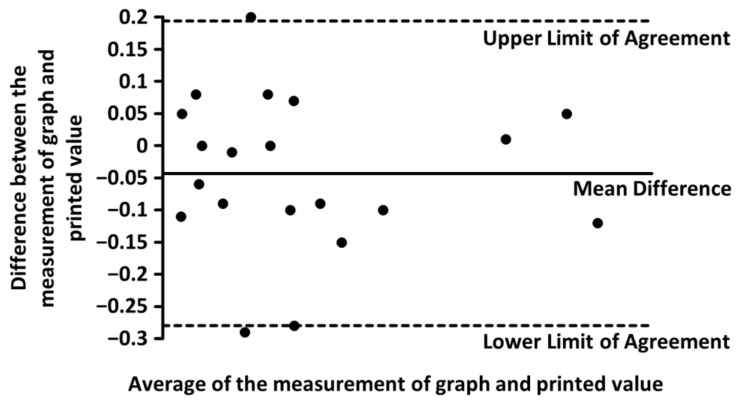
Bland-Altman plot showing average difference of −0.043 ± 0.24 (mean ± limits of agreement) between our measurement of graph and the printed values.

For the precision of the measurements in the published graphs, we used eight studies with 20 data points, where the results were both graphed and printed in text [3,4,5,10,11,12,13,14]. After the measurements of graphs, we compared those results with the data presented in text (Table 1) and checked the distribution of the differences. The Shapiro-Wilk test did not show a significant departure from normality, *W*(20) = 0.96, *p* < 0.05. Then, we created a Bland-Altman plot, which did not reveal significant differences between our measurements and the printed values (Table 1, Figure 2).

For the data comparison among the studies, we ran ANOVA or the analysis of variance. Since we were not provided with the raw data, it is necessary to briefly describe the calculations when performing ANOVA. ANOVA can be split into the so-called between-group sum of squares that is divided by its degrees of freedom, and the within-group sum of squares that is also divided by its degrees of freedom. Furthermore, a ratio of the first to the second is taken to obtain *F* statistics. The *F* statistics follow the *F* distribution. The significance of the *F* statistics (*p* value) was calculated according to the degrees of freedom of the numerator and the denominator of the ratio. A detailed description of the ANOVA can be found in a statistical textbook, e.g., Armitage et al. [15]. To assess the variability of the mean amount of LCT, we ran regression analysis. The whole analysis was performed in Microsoft Excel (2010).

To assess possible bias of our estimation of the means, we also ran a jackknife procedure (Table 2). The amount of bias was negligible in all of the five comparisons (male Sprague–Dawley and Wistar rat LCT, both stained with MT or PSR, and female Sprague–Dawley rat LCT stained with PSR).

#### 2.3.1. ANOVA—The Denominator

Because we were not provided with raw data to each of the analyzed studies to run a statistical analysis straightforwardly, we first calculated variances (*s^2^*) of the amount of connective tissue in the studied rat groups by squaring the standard deviation (*s*), which is usually calculated according to the following formula:

(1) $$s=\sqrt{\frac{\sum {\left(x_{i}-\bar{x}\right)}^{2}}{n-1}},$$

where *x_i_* is the measured value, $\bar{x}$ is the group mean of the measured value, and *n* is the number of observations. If the variability was described in terms of standard error (instead of standard deviation), we multiplied the standard error by the square root of the respective sample size. Then, to run the ANOVA, we multiplied each of the group variances with appropriate degrees of freedom (*n* − 1) to obtain the sum of squares about the group mean (*SSqM*), which is the numerator under the square root of the previous formula:

(2) $$SSqM=\sum {\left(x_{i}-\bar{x}\right)}^{2}.$$

After summing all the *SSqM* values for the compared rat groups, we obtained the total sum of squares for the groups (*SSqWithin*), which we divided with appropriate degrees of freedom:

(3) $$InGroupVariance=\frac{SSqWithin}{n-k},$$

to calculate the variance of the amount of connective tissue within the compared rat groups. In this formula, *n* stands for the total number of rats within all the compared rat groups, and *k* stands for the number of compared rat groups. The *InGroupVariance* is the denominator of the *F* ratio of ANOVA.

#### 2.3.2. ANOVA—The Numerator

For the calculation of the numerator of the ANOVA *F* ratio, first, we calculated the number weighted pooled mean ($\bar{x}$*_pooled_*) of the compared rat groups:

(4) $${\bar{x}}_{pooled}=\frac{\sum n_{k}{\bar{x}}_{k}}{n},$$

where *n_k_* represents the number of rats in the *k*th group, ${\bar{x}}_{k}$ the *k*th group mean, and *n* the total number of compared rats. Next, we calculated the sum of squares about the pooled mean of each of the rat groups according to:

(5) $$SSqOut={\left({\bar{x}}_{k}-{\bar{x}}_{pooled}\right)}^{2}\times n_{k},$$

where $\bar{x}$*_k_* stands for group mean and *n_k_* is the number of rats in the *kth* group. Then, we summed the *SSqOut* and calculated the between-group variance (*OutGroupVariance*) as follows:

(6) $$OutGroupVariance=\frac{\sum SSqOut}{k-1},$$

where *k* denotes the number of compared rat groups. Finally, we calculated the ratio of *OutGroupVariance* to InGroupVariance, which provided us with the *F* statistics.

#### 2.3.3. Detection of Significant Results

For the detection of significant differences among individual studies, after the ANOVA test, we estimated the range of the mean amount of LCT in the respective study within two standard errors of the means (not 1.96 standard error, as would be done, e.g., in the *t* test). This estimation resulted in intervals that covered 95.44% of the LCT values of each examined group (provided the distribution of the variable mean amount of connective tissue was approximately normal). Therefore, any two such intervals, which are drawn on the same axis and do not overlap, show a statistically significant difference in the parameter between compared groups, which are represented by the drawn intervals, at the level of significance *α* = 0.05 (or 0.0466). In other words, they are significantly different (*p* < 0.05).

## 3. Results

### 3.1. Literature Search

For the estimation of the amount of LCT in healthy Sprague–Dawley and Wistar rats, we ran a Medline search according to Figure 1. Out of the initial 778 studies, where the liver samples were stained either with MT or PSR, in 158 studies, the amount of LCT was shown as a percentage. However, we were able to find both the value of the mean and both the measure of variability of the presented data only in 61 studies; where the data were graphed, we included only the studies with the graphs of sufficient resolution and with clearly visible error bars. Therefore, out of the 57 remaining studies [3,4,11,12,13,14,16,17,18,19,20,21,22,23,24,25,26,27,28,29,30,31,32,33,34,35,36,37,38,39,40,41,42,43,44,45,46,47,48,49,50,51,52,53,54,55,56,57,58,59,60,61,62,63,64], we obtained data for 74 healthy rat groups (we additionally excluded ten rat groups from six studies [4,5,6,7,8,9], details are shown in Figure 1). The datasets analyzed during the current study are available in the GitHub repository, https://github.com/PM-LFP/Biased-assessment-of-rat-liver-fibrosis (accessed on 29 June 2023).

As can be seen in Table 3, the search revealed the mean amount of the rat LCT to yield in male Wistar rats from 0.51% to 5.16% when stained with MT stain and from 0.12% to 5.12% when stained with PSR stain. In male Sprague–Dawley rats, the amount of connective tissue varied from 0.55% to 9.32% when stained with MT and from 0.10% to 17.02% when stained with PSR. For female Wistar rats, our search found only one study [4], where the mean amount of PSR-stained LCT was 0.71%. In female Sprague–Dawley rats, our search revealed one study [17], where the LCT was stained with MT—the mean amount of the connective tissue was 2.08%. In the PSR-stained female Sprague–Dawley rat liver sections, the mean amount of connective tissue varied from 0.64% to 2.34%.

### 3.2. Up to 170-Fold Differences in the Amount of Healthy Rat Liver Connective Tissue

**Figure 3:**
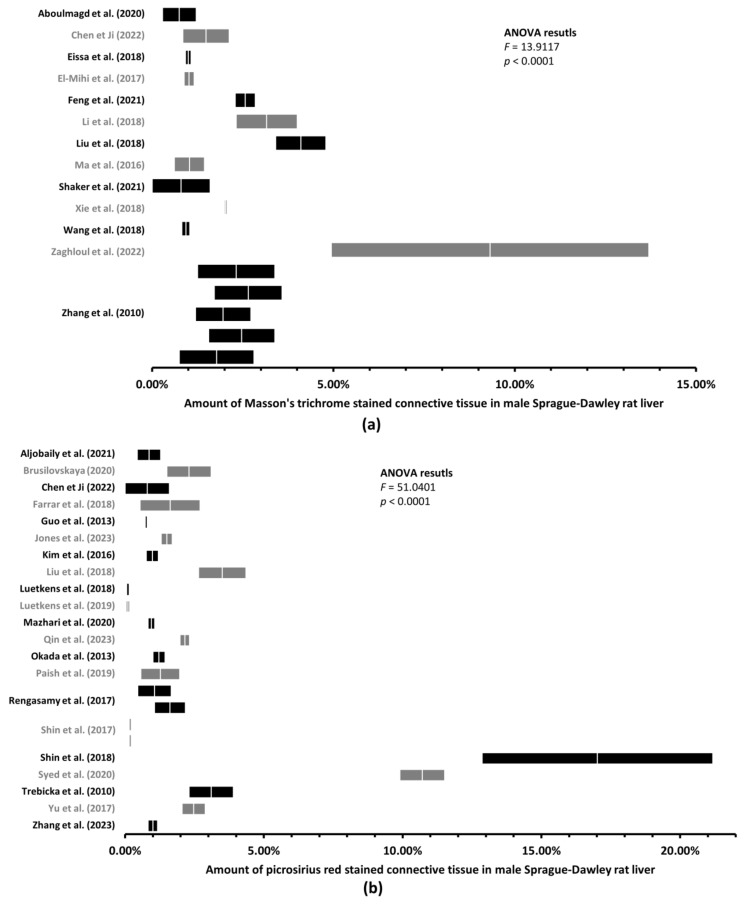
Mean amount of liver connective tissue in healthy male Sprague–Dawley rats. Graphs (mean ± two standard errors) of the amount of liver connective tissue quantified on histological sections stained with Masson’s trichrome [13,14,19,26,29,30,33,38,54,56,57,58,63] (**a**) or picrosirius red [4,13,21,25,27,32,34,36,39,42,43,44,48,49,50,52,55,58,60,62,64] (**b**) revealed significant differences. Multiple graphs within one study represents multiple rat groups within the study.

**Figure 4:**
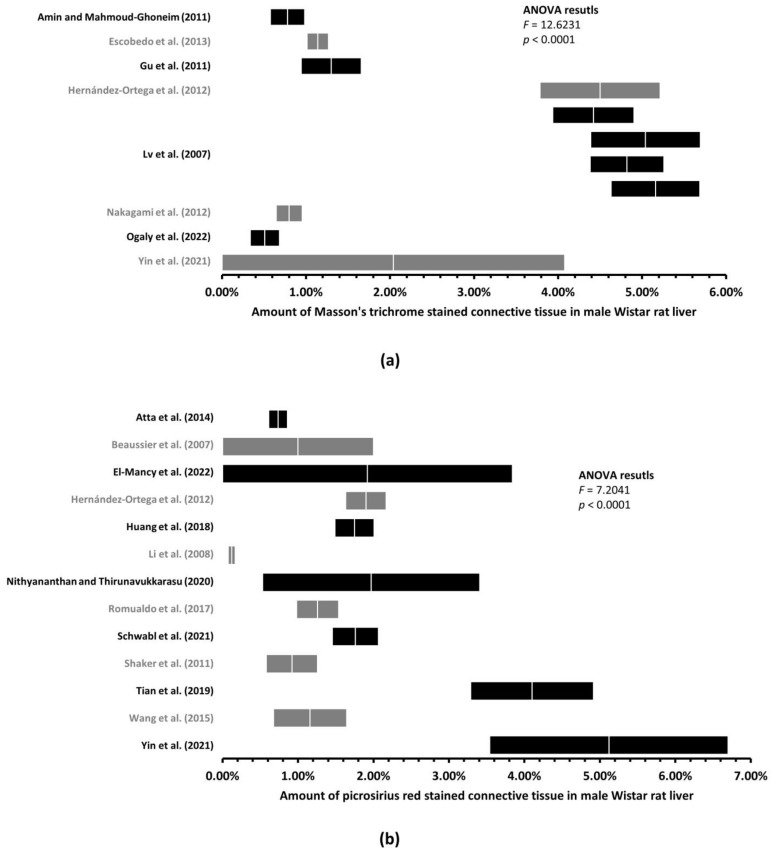
Mean amount of liver connective tissue in healthy male Wistar rats. Graphs (mean ± two standard errors) of the amount of liver connective tissue quantified on histological sections stained with Masson’s trichrome [6,11,12,24,31,40,41,61] (**a**) or picrosirius red [12,20,22,23,24,28,35,37,45,46,47,51,59] (**b**) revealed significant differences. Multiple graphs within one study represents multiple rat groups within the study.

First, we performed ANOVA in five groups of rats: male Sprague–Dawley rat liver sections stained with MT and PSR, male Wistar rat liver sections stained with MT and PSR, and female Sprague–Dawley rat liver sections stained with PSR (Figure 3 and Figure 4). By calculating the *F* statistics, we found significant differences in the amount of LCT among all the male rat groups (Figure 3) at a level of significance less than 0.0001 (this would also yield significance after correcting for multiple comparisons using Bonferroni correction), and among female rat groups stained with PSR (*p* = 0.0013, Figure 4).

**Figure 5:**
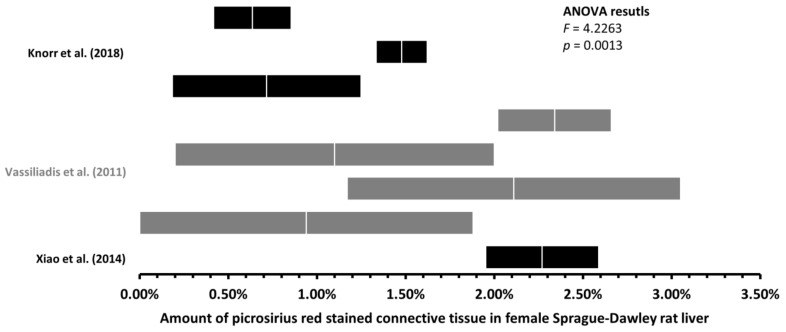
Mean amount of liver connective tissue in healthy female Sprague–Dawley rats. Graph (mean ± two standard errors) of the amount of liver connective tissue quantified on histological sections stained with picrosirius [17,18,53] red revealed significant differences. Multiple graphs within one study represents multiple rat groups within the study.

Second, to find significant differences in the amount of rat LCT, we compared the respective ranges of estimates for each of the mean amounts of connective tissue, with the upper and lower limits being plus or minus two standard errors of the mean (see Section 2). We found significant differences in the mean amount of LCT among the healthy male Sprague–Dawley rats when stained with MT (Figure 3a) and with PSR (Figure 3b), and among male Wistar rats when stained with MT (Figure 4a) and with PSR (Figure 4b). As shown in Table 2, some of the differences in the mean amount of connective tissue were up to 170-fold different (0.10% to 17.02%). Furthermore, when the differences in the mean amount of connective tissue among female Sprague–Dawley rat liver sections stained with PSR were assessed, we found significant differences even within individual studies (e.g., 0.64% vs. 1.48%; 0.91% vs. 2.34%), where more experiments were conducted following the same protocol [17,18] (Figure 5).

### 3.3. With More Connective Tissue, Tendency of the Variability of the Estimated Amount of Connective Tissue to Decrease

Another finding of our analysis was the higher variability of the estimated mean amount of rat LCT in terms of standard deviation. To assess the relative variability, we calculated the coefficient of variation (CV) of the mean amount of LCT, which is the standard deviation of the mean divided by the mean. The calculated value of the CV ranged from 0.02 to 1.04. In other words, when comparing the amount of LCT among groups of rats, we can expect the value of the mean to vary up to 104% of its value. However, on the average we could expect the variability of the estimated average to range from 20% to 30% of the estimated average (95% confidence interval of the average CV).

**Figure 6:**
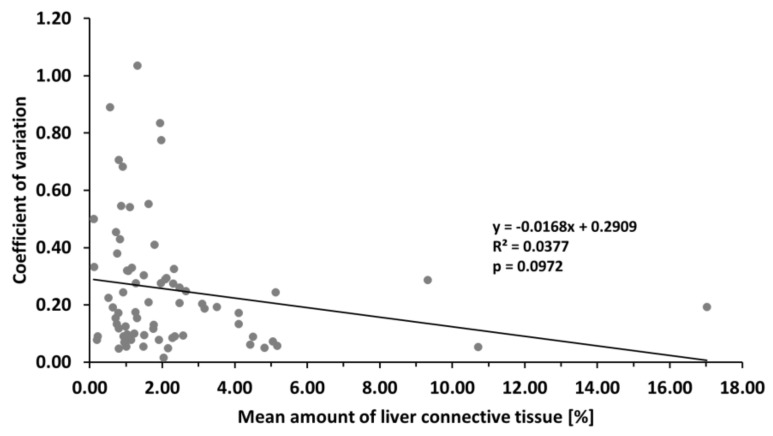
Regression analysis of the mean amount of liver connective tissue and the coefficient of variation. Based on the data retrieved from the eligible studies, the regression line was fitted according to the least squares method. Therefore, with more connective tissue in the liver, there is a tendency toward lowering the variability of the estimated amount of connective tissue. However, the actual changes in the value of the mean amount of connective tissue can explain approximately 3.8% (*R*^2^ = 0.0377) of the observed variability.

To further analyze these studies and push our analysis further, we ran a regression analysis to explore the association of CV to the value of the mean, and we did not find a significant negative association (*F* = 2.82, *p* = 0.0972). However, according to the least squares method, we fitted a regression line y = −0.0168x + 0.2909 (Figure 6). Therefore, with more connective tissue in the liver, there is a tendency toward lowering the variability of the estimated amount of connective tissue. However, the actual value of the mean amount of connective tissue can explain approximately 3.8% (*R^2^* = 0.0377) of the observed variability. On the other hand, as seen in Figure 6, nonhomogenous variance can be observed in the data (heteroscedasticity). Therefore, logarithmic transformation of the data should be done prior to the modeling or predicting the variability of the mean amount of connective tissue, but more significantly, the data we used are probably biased (see Section 4).

## 4. Discussion

Our analysis revealed significant differences in the amount of connective tissue in healthy rat livers among all the compared rat groups. The difference in the mean amount of LCT was up to 170 times different (0.10% to 17.02%), and significant differences were found even within one study.

### 4.1. Biased Sampling and Quantification of Rat Liver Connective Tissue

The significant variability of the amount of the healthy rat LCT could stem from unstandardized and biased sampling of rat liver samples. Generally, details regarding the sampling of either tissue blocks or histological sections or about the sampling of microphotographs provided by the authors varied vastly. Unfortunately, in most of the analyzed studies, the description of the sampling methods prevented the readers from repeating both the sampling and the histological quantification of fibrosis. When studying sampling design, in 49 (86%) of the studies, neither the original location of the tissue sample nor the tissue sampling process was specified to the extent for possible replication of the assessment of liver fibrosis. Only in seven of them (14%) was at least the original liver lobe of the sample specified [15,16,17,18,19,20,65]. When sampling microphotographs for quantification of connective tissue, the authors usually provided data regarding the hardware (type of microscope and camera with its resolution), software, and the number of photographs per slide used. Unfortunately, any measure of the reliability and validity that would justify the proposed sampling scheme and quantification is missing, e.g., graphs of moving averages of the estimated amount of connective tissue between photographs or tissue blocks, or the coefficient of error of the quantification of the amount of connective tissue. Therefore, none of the analyzed studies met recommended standards for reliable quantification, such as stereological unbiased sampling and quantification [66]. Only after the adjustment of the sampling scheme were we be able to decide whether the observed variability of up to 100% in the amount of LCT is a characteristic of the liver, and consequently, how much variability we can expect.

The unstandardized and biased methodology of the LCT quantifications also spans research in other animal models, and significant differences in the mean amount of LCT can also be found in studies of healthy mice (mean amount of LCT 0% to 7%) [67,68,69,70], hamsters (mean amount of LCT 0.13% to 5%) [71,72], rabbits (mean amount of LCT 0.03% to 6.23%) [73,74] and cows (mean amount of LCT 0% to 6.17%) [75,76]. On the other hand, to date, we have found only six studies of laboratory animals that fulfill the criteria of unbiased sampling and, hence, provided us with an objective measure of the amount of LCT in healthy individuals [77,78,79,80,81,82] (Table 4). Even though we also found differences among these studies, we were not able to assess their significance due to the missing measure of variability (standard deviation or error) in most of them.

### 4.2. Reliability and Validity of the Assessment of Liver Fibrosis

Although the staging of the liver biopsy is still necessary for the assessment of liver fibrosis, the usual qualitative (or semiquantitative) evaluation has several limitations that can be broadly summarized into three categories: quality of specimen, representativeness of specimen, and validity and reliability of the assessment.

First, for reliable staging of the liver biopsy, a sufficient number of complete portal tracts should be visible. It was suggested that six to eight portal triads are sufficient [83], which equates to at least 1.6 cm in length of the biopsy specimen [84]. However, it has been recommended to only assess the biopsy specimens with at least 11 complete portal tracts [85] or specimens at least 2.5 cm in length [86]. Assessment of fibrosis on such a kind of specimen is associated with an improvement in correct classification of the specimen.

Second, a human liver biopsy represents approximately 1/50,000th of the organs. Therefore, when assessing two biopsies of different liver lobes, 80% disagreement (33 of 41 biopsy pairs) in the fibrosis stage can be expected [87]. Furthermore, when assessing two neighboring liver biopsies of the same liver lobe, one or more fibrosis stage differences on a five-grade scale can be expected in 41% of cases [88]. It has been proven that these differences cannot be attributed to intra-observer variation [89].

Third, in the qualitative assessment of liver fibrosis, inter-observer and intra-observer variability and the resulting subjectivity of the fibrosis assessment disqualifies the data for observational study use and for the noninvasive methods of liver fibrosis evaluation. Although a blinded consensus biopsy review has been proposed in targeting the subjectivity of the assessment [90], a quantitative approach would provide us with a more sensitive differentiation of the fibrosis state [91].

### 4.3. Fibrosis Stage Is Not a Continuous Variable

Too often, when reporting and comparing experimental outcomes in terms of liver fibrosis stages, the data are treated as continuous variables [16,18,21,22,92]. Fibrosis stage is a category that describes both the amount of LCT quantified on the liver biopsy and the architecture of the connective tissue. These categories can be ordered according to their proximity to liver cirrhosis. Therefore, first, it does not provide us only with a measure of quantity, but also with the description of quality—forming of septa, bridging, central or portal fibrosis. Second, when we are describing ordinal variables, it does not matter whether we label the respective category with a number, letter, or word. Hence, we cannot calculate an average from three liver biopsies of stage “none”, 10 biopsies of stage “mild”, 12 biopsies of stage “moderate”, four biopsies of stage “severe”, and two biopsies of stage “cirrhosis”. Furthermore, counterintuitively, the amount of connective tissue in the fibrosis stage should be regarded as a categorical variable since it has been shown that the amount of connective tissue quantified from the liver biopsy can be the same between Ishak stages 0 and 5 [93], and Metavir stages F0 and F4 [94]. Third, this measure of quantity itself describes a nonlinear trend in the amount of LCT between the respective stages of liver fibrosis [94]. Fourth, if we were to dismiss all the objections proposed thus far and would calculate the average out of fibrosis stages, what would be the interpretation of average Metavir fibrosis stage 2.4 (note the missing letter F) with standard deviation of 0.5 [95], and what would be the difference to stage, e.g., 2.9 or 2.2?

### 4.4. Practical Recommendations

For experiments in animal models, practical guidelines for unbiased sampling and quantification have already been proposed [96], even specifically for rat livers [66]. At first, a pilot study should be done to assess the expected sampling strategy according to the following steps [97]. Unbiased quantification of LCT would comprise the first measurement of fresh liver volume [96]. This step would not be necessary if the proportion of the sampled tissue blocks to the whole organ is known. Second, depending on the size of the sampled organ, the liver should either be cut into slices; the slices for further analysis should be selected by independent random sampling or systematic uniform random sampling, or the slices may be arranged smoothly using a smooth fractionator and then selected [66]. If the slices cannot be processed as a whole, the slices should be cut exhaustively into tissue blocks and then tissue blocks should be selected using systematic uniform sampling [97]. Usually, between five and ten slices (or tissue blocks) per organ is sufficient. Third, tissue slices or tissue blocks should be cut exhaustively on a suitable number of systematic uniform random sections (usually 12 per tissue slice/block is sufficient, but see fifth step). Fourth, sampling of the microphotographs (or fields of view) should preferably be followed by quantification of connective tissue using a point grid and point counting method or by image analysis. Fifth, according to the value of the coefficient of error [98]—a value of approximately 10% is recommended—and the analysis of the moving average, our sampling scheme should be adjusted at each level of sampling. Therefore, if the coefficient of error is too large, denser sampling should be applied, starting from the last step and gradually advancing to the first step. Finally, after adjusting our sampling scheme, the unbiased quantification of LCT can be performed.

### 4.5. Clinical Application

When scoring human liver fibrosis, the most convenient application at hand in human medicine would be taking at least five liver biopsies, followed by the recommended procedure, starting from step three. Unfortunately, both this recommendation and our experimental recommendations usually cannot be adhered to in human medicine due to ethical restrictions. Therefore, completing a proof-of-concept study on animal model random samples of livers of different stages of liver fibrosis (at least six individuals per stage) combined with liver elastography and serum markers of liver fibrosis assessment would be necessary. First, the assessment of liver stiffness would assume taking multiple measurements of liver tissue stiffness from different foci in standardized fashion. Second, blood samples should be taken to further assess noninvasive scoring of liver fibrosis (e.g., APRI score and FIB-4 score). Third, quantification of liver fibrosis according to the recommendation in the previous section should be done. Fourth, the correlation analysis with AUROC analysis of the assessed parameters should provide us with the best parameters that could be studied in human samples.

Then, following a similar scheme, human autopsy random samples of livers combined with perimortem liver elastography assessment should be performed combined with serological markers of liver fibrosis. Hopefully, both the unbiased sampling of liver stiffness and the serological markers of liver fibrosis could be used for distinguishing even early stages of liver fibrosis with personalized predictions for treatment and its outcomes.

### 4.6. Minimum Detectable Effect

To plan a pilot study, we can use our biased data to get an idea of detectable changes in LCT and the sample size needed to detect the changes. For the minimal detectable change, we run a minimum detectable effect analysis according to Ahn et al. [99]; and for the sample size needed to detect respective fold changes in LCT, we run an analysis according to Vittinghoff et al. [100]. The results are summarized in Table 5. The minimal detectable change in the amount of LCT ranged between a 0.51% and 0.81% increase of LCT. The sample size decreased exponentially with the increase in the respective fold change.

### 4.7. Limitations

Although we provide the step-by-step procedure for unbiased quantification of LCT in experimental settings and its translation into clinics, our study has some limitations. We did not further subdivide our analysis according to the age of the rats. Since the amount of LCT increases with age in male rats [79], this could possibly introduce a bias into our study. Differences in the sample sizes in the analyzed studies put different weights on the data, and this was not addressed, nor were the studies with small sample sizes (e.g., two, three, four, or five rats) excluded. This could influence the variability measure (standard error or standard deviation). The immunological incompatibility of rat models hinders potential clinical translation of fibrosis quantification in the case of, e.g., viral hepatitides. Even the larger amount of LCT in healthy liver in rat when compared to human liver has to be accounted for [101,102].

## 5. Conclusions

We found significant differences in the amount of connective tissue in healthy rat livers among all the compared rat groups. The difference in the mean amount of LCT was up to 170 times different (0.10% to 17.02%), and significant differences were found even within one study. The 95% confidence interval of the estimated mean amount of LCT can be expected to vary up to 104% of the value of the estimated mean, but the increase in the amount of LCT was followed by a decrease in variability (i.e., narrowing of standard deviation). The observed significant variability may be due to biased histological sampling and subsequent quantification. This bias can be prevented, however, by following the guidelines for the unbiased quantification of rat LCT. In particular, independent random sampling, systematic uniform random sampling, or the smooth fractionator should be used for tissue sampling, adjusted according to the analysis of the coefficient of error and moving average. Then, quantification should be performed following the point counting method. Despite some limitations, in this way, not only could the ongoing reproducibility crisis be addressed, but such unbiased data could also eventually be used for the validation of noninvasive (or minimally invasive) methods of the assessment of early stages of liver fibrosis.

## Figures and Tables

**Table 1:** Differences between our measurements of graphs and printed values.

Parameter	Measurement of Graph	Printed Value
N	20	
Mean	1.40%	1.44%
Standard Deviation	1.26%	1.26%
Mean Difference	−0.043 ± 0.24%	
+/− Limits of Agreement		

**Table 2:** Jackknife procedure of the assessment of bias.

Rat Strain	Sex	Staining Method	N	Mean Amount of Liver Connective Tissue		
				Pooled Mean	Jackknife Estimate of Pooled Mean	Bias
Wistar	M	Masson’s trichrome	11	2.76%	3.04%	0.00%
		Picrosirius red	13	1.76%	1.91%	0.00%
	F	Masson’s trichrome	No data			
		Picrosirius red	1	One study—Mannheimer et al. [3], 0.71%		
Sprague–Dawley	M	Masson’s trichrome	17	2.30%	2.45%	0.00%
		Picrosirius red	23	2.41%	2.52%	0.00%
	F	Masson’s trichrome	1	One study—Zhang et al. [16], 2.08%		
		Picrosirius red	8	1.45%	1.65%	0.00%

**Table 3:** Amount of liver connective tissue in healthy rats.

Rat Strain	Sex	Staining Method	Number of Rat Groups	Mean Amount of Liver Connective Tissue			
				Minimum	Maximum	Pooled Mean	Pooled Standard Error
Wistar	M	Masson’s trichrome	11	0.51%	5.16%	2.76%	0.27%
		Picrosirius red	13	0.12%	5.12%	1.76%	0.34%
	F	Masson’s trichrome	No data				
		Picrosirius red	1			0.71%	0.11%
				One study—Mannheimer et al. [3]			
Sprague–Dawley	M	Masson’s trichrome	17	0.55%	9.32%	2.30%	0.40%
		Picrosirius red	23	0.10%	17.02%	2.41%	0.29%
	F	Masson’s trichrome	1			2.08%	0.54%
				One study—Zhang et al. [16]			
		Picrosirius red	8	0.64%	2.34%	1.45%	0.27%

**Table 4:** Objectively quantified amount of liver connective tissue in laboratory animals ^1^.

Laboratory Animal	Sex	Staining Method	Amount of Liver Connective Tissue (Mean ± SD)	Reference
Mouse	M	Picrosirius red	1.45 ± 0.07%	Clapper et al. [77]
Rat	M	Picrosirius red	1.50%	Fallowfield et al. [78]
	M	Picrosirius red	0.50%	Hoy et al. [79]
	M	Picrosirius red	2.0 ± 0.3%	Marcos et al. [80]
	M	Picrosirius red	3.2 ± 0.2%	
	F	Picrosirius red	2.00%	Marcos a Correia-Gomes [81]
Pig	M	Aniline blue	4.7 ± 2.4%	Mik et al. [82]
	F	Aniline blue	3.6 ± 2.2%	

^1^ Modified from Mik P. Porcine liver anatomy applied in biomedicine [Doctoral thesis]. Plzen, Czech Republic: Charles University, 2021. Available from: https://dspace.cuni.cz/handle/20.500.11956/147917 (accessed on 29 June 2023).

**Table 5:** Minimum detectable increase (MDI) in liver connective tissue (LCT) and sample size (Size) needed to detect fold increase in LCT in Wistar and Sprague–Dawley (SD) rats.

Rat Strain	Sex	Staining Method	Mean Amount of LCT	MDI in LCT	Expected Increase in LCT/Minimal Sample Size for the Detection of Such Increase					
					1.25-Fold	Size	1.5-Fold	Size	2-Fold	Size
Wistar	M	Masson’s trichrome	2.76%	0.59%	3.45%	19	4.14%	5	5.52%	1
	M	Picrosirius red	1.76%	0.81%	2.20%	48	2.64%	12	3.52%	3
SD	M	Masson’s trichrome	2.30%	0.74%	2.88%	28	3.45%	7	4.60%	2
	M	Picrosirius red	2.41%	0.51%	3.01%	25	3.62%	6	4.82%	2
	F	Picrosirius red	1.45%	0.69%	1.81%	70	2.18%	18	2.90%	4

## Data Availability

The datasets analyzed during the current study are available in the GitHub repository, https://github.com/PM-LFP/Biased-assessment-of-rat-liver-fibrosis (accessed on 29 June 2023).

## Supplementary Materials

The following supporting information can be downloaded at: https://www.mdpi.com/article/10.3390/jcm12155072/s1, Table S1: Commonly used scoring systems for the assessment of liver fibrosis [103,104,105,106,107,108].
