# Supplementary material for: Biased Quantification of Rat Liver Fibrosis—Meta-Analysis with Practical Recommendations and Clinical Implications
Source: J Clin Med. 2023 Aug 1;12(15):5072. doi: 10.3390/jcm12155072 (PMC10420125; doi:10.3390/jcm12155072)
Supplement: Supplementary file 1 [file jcm-12-05072-s001.zip › jcm-2508209-supplementary.pdf]

Supporting Table S1 for:

Biased Quantification of Rat Liver Fibrosis—Meta-Analysis with Practical Recommendations and Clinical Implications

Patrik Mik, Katsiaryna Barannikava and Polina Surkova

Supporting Table S1 Commonly used scoring systems for the assessment of liver fibrosis.

Scoring system	Stage	Description	Reference
Histological activity index	A	No fibrosis	Knodell et al. [103]
	B	Fibrous portal expansions	
	C	Bridging fibrosis (portal or portal-central linkage)	
	D	Cirrhosis	
Scheuer	0	None	Scheuer [104]
	1	Enlarged, fibrotic portal tracts	
	2	Periportal or portal-portal septa but intact architecture	
	3	Fibrosis with architectural distortion but no obvious cirrhosis	
	4	Probable or definite cirrhosis	
Batts and Ludwig	0	Normal connective tissue (No fibrosis)	Batts and Ludwig [105]
	1	Fibrous portal expansion (Portal fibrosis)	
	2	Periportal or rare portal-portal septa (Periportal fibrosis)	
	3	Fribrous septa with architectural distortion; no obvious cirrhosis (Septal fibrosis)	
	4	Cirrhosis	
Ishak	0	No fibrosis	Ishak et al. [106]
	1	Fibrous expansions of some portal areas, with or without short fibrous septa	
	2	Fibrous expansions of most portal areas, with or without short fibrous septa	
	3	Fibrous expansions of most portal areas with occasional portal to portal (P-P) bridging	
	4	Fibrous expansions of portal areas with marked bridging (portal to portal (P-P) as well as portal to central (P-C))	
	5	Marked bridging (P-P) and/or (P-C) with occasional nodules (incomplete cirrhosis)	
	6	Cirrhosis, probable or definite	
Metavir	F0	No fibrosis	Bedossa and Poynard [107]
	F1	Portal fibrosis without septa	
	F2	Portal fibrosis with rare septa	
	F3	Numerous septa without cirrhosis	
	F4	Cirrhosis	
Kleiner	0	None	Kleiner et al. [108]
	1	Perisinusoidal or periportal	
	1A	Mild, zone 3, perisinusoidal	
	1B	Moderate, zone 3, perisinusoidal	
	1C	Portal/periportal	
	2	Perisinusoidal and portal/periportal	
	3	Bridging fibrosis	
	4	Cirrhosis	

Bibliography

103. Knodell, R.G.; Ishak, K.G.; Black, W.C.; Chen, T.S.; Craig, R.; Kaplowitz, N.; Kiernan, T.W.; Wollman, J. Formulation and Application of a Numerical Scoring System for Assessing Histological Activity in Asymptomatic Chronic Active Hepatitis. *Hepatology* **1981**, *1*, 431–435, doi:10.1002/hep.1840010511.
104. Scheuer, P.J. Classification of Chronic Viral Hepatitis: A Need for Reassessment. *J. Hepatol.* **1991**, *13*, 372–374.
105. Batts, K.P.; Ludwig, J. Chronic Hepatitis. An Update on Terminology and Reporting. *Am. J. Surg. Pathol.* **1995**, *19*, 1409–1417.
106. Ishak, K.; Baptista, A.; Bianchi, L.; Callea, F.; De Groote, J.; Gudat, F.; Denk, H.; Desmet, V.; Korb, G.; MacSween, R.N. Histological Grading and Staging of Chronic Hepatitis. *J. Hepatol.* **1995**, *22*, 696–699.
107. Bedossa, P.; Poynard, T. An Algorithm for the Grading of Activity in Chronic Hepatitis C. The METAVIR Cooperative Study Group. *Hepatol. Baltim. Md* **1996**, *24*, 289–293, doi:10.1002/hep.510240201.
108. Kleiner, D.E.; Brunt, E.M.; Natta, M.V.; Behling, C.; Contos, M.J.; Cummings, O.W.; Ferrell, L.D.; Liu, Y.-C.; Torbenson, M.S.; Unalp-Arida, A.; et al. Design and Validation of a Histological Scoring System for Nonalcoholic Fatty Liver Disease. *Hepatology* **2005**, *41*, 1313–1321, doi:10.1002/hep.20701.
